# Preoperative inflammatory and immune-nutritional markers and postoperative pulmonary complications after gastric and colorectal cancer surgery: a systematic review and narrative synthesis

**DOI:** 10.3389/fsurg.2026.1850606

**Published:** 2026-07-02

**Authors:** Hongyue Zhang, Lingyun Zou, Hongjuan Fang, Hao Wang

**Affiliations:** 1 Department of General Surgery, The First People’s Hospital of Jiande, Jiande, China; 2 Department of Thoracic Surgery, Affiliated Zhongshan Hospital of Dalian University, Dalian, China

**Keywords:** colorectal cancer surgery, gastric cancer surgery, immune-nutritional markers, inflammatory biomarkers, postoperative pneumonia, postoperative pulmonary complications

## Abstract

**Background:**

Postoperative pulmonary complications (PPCs) remain a major source of morbidity after gastrointestinal cancer surgery. A range of preoperative inflammatory and immune-nutritional biomarkers have been proposed as predictors of postoperative pulmonary risk, but the available evidence remains scattered and methodologically heterogeneous. This study aimed to systematically evaluate the association between preoperative inflammatory and immune-nutritional markers and postoperative pulmonary complications after gastrointestinal cancer surgery.

**Methods:**

Following PRISMA 2020 guidelines, we systematically searched PubMed, Embase, Web of Science Core Collection, Scopus, and the Cochrane Library from database inception to 3 March 2026. Original observational studies evaluating preoperative inflammatory or immune-nutritional markers in adult patients undergoing surgery for gastrointestinal malignancies were eligible if they reported PPCs, postoperative pneumonia, or other extractable pulmonary complications. The primary outcome was PPCs, while postoperative pneumonia and other individual pulmonary complications were extracted separately when available. Due to substantial heterogeneity in pulmonary outcome definitions, biomarker selection, cutoff determination, and statistical modeling, findings were synthesized narratively.

**Results:**

Fifteen studies were included, all of which were observational in design, with overall moderate-to-high methodological quality. Across studies reporting broader PPCs and infectious pulmonary outcomes, adverse preoperative inflammatory and nutritional profiles were generally associated with increased postoperative pulmonary risk. Inflammatory markers such as neutrophil-to-lymphocyte ratio and systemic immune-inflammation index demonstrated the strongest associations in individual studies, while nutritional indicators including albumin, controlling nutritional status score, prognostic nutritional index, and geriatric nutritional risk index consistently indicated an elevated risk in patients with poorer preoperative nutritional status. Overall, the findings pointed to a recurrent pattern of combined inflammatory burden and nutritional impairment rather than reliance on a single biomarker. Because most biomarker-specific findings were supported by single studies and were reported using heterogeneous outcome definitions and analytic scales, formal pooled meta-analysis was not performed.

**Conclusions:**

Current evidence suggests that a preoperative double burden of heightened systemic inflammation and impaired nutritional status is associated with increased postoperative pulmonary risk after gastric and colorectal cancer surgery. However, the literature remains heterogeneous in outcome definitions, biomarker modeling, and effect reporting. Standardized prospective studies are needed before these markers can be reliably integrated into perioperative pulmonary risk stratification.

**Systematic Review Registration:**

https://www.crd.york.ac.uk/PROSPERO/view/CRD420261361210, identifier CRD420261361210.

## Introduction

Gastrointestinal malignancies continue to impose a substantial global health burden, with colorectal and gastric cancers remaining among the most frequently diagnosed cancers and major causes of cancer-related death worldwide (1). For patients with resectable disease, surgery remains the cornerstone of curative-intent treatment. However, postoperative complications continue to shape both perioperative recovery and the overall burden of care. In contemporary gastric cancer surgery, pulmonary complications remain among the major adverse postoperative events and contribute meaningfully to prolonged recovery, escalation of care, and adverse clinical outcomes (2). More broadly, postoperative pulmonary complications (PPCs) remain a persistent perioperative challenge, and recent evidence suggests that although multiple risk prediction models have been proposed, their reproducibility and clinical utility remain inconsistent (3).

This ongoing clinical problem has renewed interest in preoperative host-related markers that are inexpensive, readily available, and biologically plausible. In patients undergoing major gastrointestinal cancer surgery, systemic inflammation, immune dysregulation, and impaired nutritional reserve often coexist rather than occur in isolation. The 2025 ESPEN guideline on clinical nutrition in surgery emphasizes that malnutrition is a major determinant of postoperative outcome and that perioperative nutritional assessment is particularly relevant in major cancer surgery (4). Within this context, preoperative inflammatory and immune-nutritional biomarkers derived from routine blood tests offer an attractive means of capturing the combined burden of inflammatory activation and reduced physiological reserve before surgery.

Several gastrointestinal surgical oncology studies have begun to examine this issue more directly. In gastric cancer, elevated neutrophil-to-lymphocyte ratio (NLR) and higher systemic inflammation score (SIS) have been associated with postoperative pneumonia after gastrectomy (5, 6). In colorectal cancer surgery, poorer preoperative nutritional status measured by the controlling nutritional status (CONUT) score, prognostic nutritional index (PNI), and geriatric nutritional risk index (GNRI) has also been linked to postoperative pulmonary morbidity or pulmonary infection (7, 8). Taken together, these studies suggest that postoperative pulmonary risk may reflect not a single abnormal biomarker, but a broader preoperative imbalance across inflammatory and nutritional domains.

Despite growing interest, the current literature remains difficult to interpret as a coherent body of evidence. Studies differ substantially in tumour site, surgical population, pulmonary endpoint definition, and analytic strategy. Some evaluate broader PPC composites, whereas others focus specifically on postoperative pneumonia or postoperative pulmonary infection. Biomarkers are variably modelled as continuous variables, dichotomized cutoffs, or ordered categories, further limiting direct comparability across studies. These differences have made it difficult to determine whether preoperative inflammatory and immune-nutritional markers provide a reproducible clinical signal across gastrointestinal cancer surgery. We therefore conducted a systematic review and narrative synthesis to evaluate the association between preoperative inflammatory and immune-nutritional markers and postoperative pulmonary complications after gastrointestinal cancer surgery. PPCs were treated as the primary outcome framework, while postoperative pneumonia and other individual pulmonary complications were extracted separately when reported. Our aim was to clarify which signals appear consistent across the current evidence base and where important uncertainty remains.

## Methods

### Protocol registration and reporting standard

This study was designed as a systematic review with narrative synthesis and reported in accordance with the PRISMA 2020 statement (9). The review protocol was prospectively registered in PROSPERO (CRD420261361210).

### Literature search

PubMed, Embase, Web of Science Core Collection, Scopus, and the Cochrane Library were searched independently by two reviewers from database inception to 3 March 2026. All electronic database searches were completed on 3 March 2026, and the final database-specific record counts were used to construct the PRISMA flow diagram. The strategy combined subject headings and free-text terms related to gastrointestinal malignancies, gastrointestinal cancer surgery, preoperative inflammatory and immune-nutritional markers, and postoperative pulmonary outcomes. Search syntax was adapted for each database. Reference lists of eligible studies and relevant reviews were also screened manually for additional records, which were assessed using the same eligibility criteria as database-derived records. Full search strategies for all databases are provided in the Supplementary Material.

### Eligibility criteria

Eligible studies were original observational studies of adult patients (≥18 years) undergoing surgery for gastrointestinal malignancies that evaluated at least one preoperative inflammatory or immune-nutritional marker and reported postoperative pulmonary complications (PPCs), postoperative pneumonia, or other extractable pulmonary complications. Cohort and case-control studies were eligible. Studies had to provide extractable association data, including odds ratios (ORs), risk ratios (RRs), hazard ratios (HRs), corresponding 95% confidence intervals (CIs), or sufficient raw data for structured interpretation.

Studies were excluded if they did not involve gastrointestinal cancer surgery, did not assess preoperative markers, or failed to report pulmonary outcomes separately. We also excluded non-original publications, conference materials, preprints, theses, animal studies, and basic science studies, as well as non-English reports and articles without accessible full text. For potentially overlapping cohorts, the most informative dataset was retained based on sample size, completeness of reporting, and relevance to the review question.

### Outcome framework

The primary outcome was postoperative pulmonary complications after gastrointestinal cancer surgery. PPCs were defined according to the original studies and could include composite pulmonary events such as postoperative pneumonia, atelectasis, respiratory failure, pleural effusion, pneumothorax, prolonged mechanical ventilation, reintubation, acute respiratory distress syndrome, or other clearly pulmonary postoperative adverse events. Postoperative pneumonia was prespecified as an important secondary pulmonary outcome and extracted separately whenever available. Other individual pulmonary complications were also recorded when clearly defined and extractable. When PPC definitions varied across studies, the original study definitions were retained.

### Study selection

Two reviewers independently screened records in two stages. After duplicate removal, titles and abstracts were reviewed for relevance, followed by full-text assessment of potentially eligible articles against the predefined criteria. Disagreements were resolved by discussion, with third-reviewer adjudication when required.

### Data extraction

Data were extracted independently by two reviewers using a standardized form. The following items were collected: first author, publication year, country, study design, study population, surgical procedure, sample size, biomarker type, biomarker definition or cutoff, timing of biomarker measurement, outcome definition, and reported association measures.

Multivariable-adjusted estimates were prioritized when available; univariable results were recorded when adjusted analyses were not reported. If a study evaluated more than one eligible biomarker, each biomarker-specific result was extracted separately, while interpretation remained anchored to the source study. Biomarkers were considered individually rather than combined into a single overall effect across marker types.

### Quality assessment

Methodological quality was assessed independently by two reviewers using the Newcastle–Ottawa Scale (NOS; range, 0–9). Disagreements in scoring were resolved through discussion with a third reviewer when necessary.

### Synthesis strategy

Because the included studies differed substantially in tumor type, surgical approach, pulmonary outcome definition, biomarker selection, cutoff derivation, and statistical modeling, the evidence was synthesized narratively rather than pooled in a formal meta-analysis. Studies reporting broader PPCs were summarized first, followed by studies reporting postoperative pneumonia or other individually extractable pulmonary complications.

Within each outcome category, findings were summarized by biomarker, with emphasis on direction of association, retention in multivariable models, and the analytic scale used in the original study, including continuous, dichotomized, and ordinal-category approaches. For nomogram or prediction-model studies, biomarker-specific associations were interpreted only if they were separately reported or extractable. Model performance measures, such as AUC, C-index, calibration, and decision-curve analysis, were considered separately and were not treated as equivalent to independent biomarker associations. When evidence for a given biomarker was limited to a single study or reported on incompatible scales, findings were retained for structured narrative synthesis.

## Results

### Study selection and characteristics

**Figure 1:**
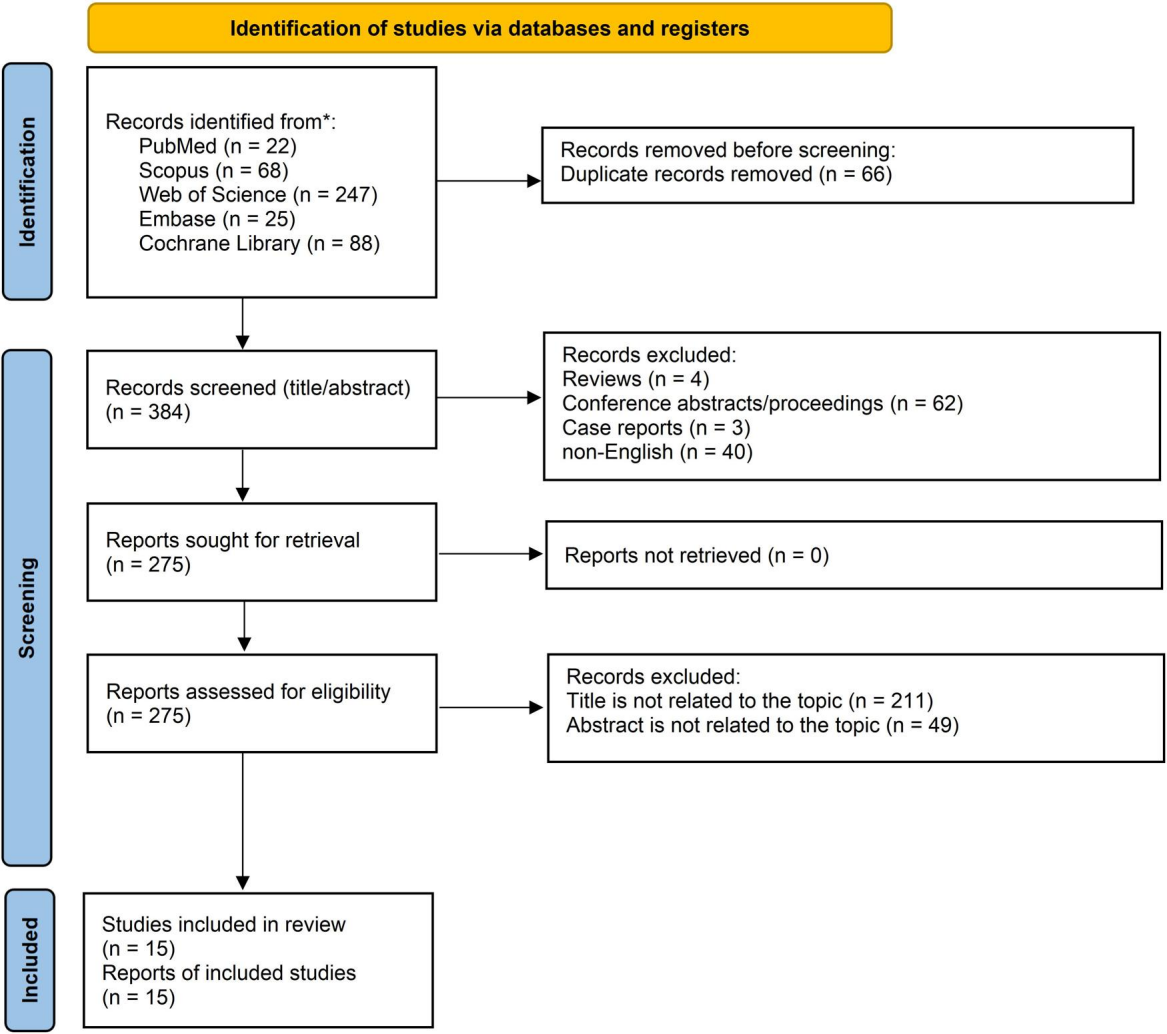
PRISMA 2020 flow diagram showing study identification, duplicate removal, title/abstract screening, full-text eligibility assessment, and final inclusion. Electronic database searches were conducted from database inception to 3 March 2026. *From: Page, M.J., McKenzie, J.E., Bossuyt, P.M., Boutron, I., Hofmann, T.C., Mulrow, C.D., Shamseer, L., Tetzlaf, J.M., Akl, E.A., Brennan, S.E., et al. (2021). The PRISMA 2020 statement: an updated guideline for reporting systematic reviews. Syst Rev. Mar 29;10(1):89. doi: 10.1186/s13643-021-01626-4. PMID: 33781348; PMCID: PMC8008539.

From database inception to 3 March 2026, the electronic database search identified 450 records, including 22 from PubMed, 68 from Scopus, 247 from Web of Science, 25 from Embase, and 88 from the Cochrane Library. After removal of 66 duplicate records, 384 records underwent title and abstract screening. Of these, 275 reports were assessed in full text for eligibility, and 15 studies were ultimately included in the systematic review (Figure 1).

All included studies were observational in design. The included studies were conducted in China or Japan and mainly involved patients undergoing gastrectomy for gastric cancer or radical surgery for colorectal cancer. Sample sizes ranged from 108 to 7,130, indicating substantial variation in study scale and clinical setting across the evidence base (5–8, 10–20). Most were retrospective single-center cohort studies, whereas two were retrospective multicenter cohort studies (6, 17). The evaluated biomarkers included albumin, cholinesterase, neutrophil-to-lymphocyte ratio (NLR), systemic inflammation score (SIS), systemic immune-inflammation index (SII), red blood cell distribution width (RDW), prognostic nutritional index (PNI), platelet-to-lymphocyte ratio (PLR), systemic inflammation response index (SIRI), albumin-to-globulin ratio (AGR), controlling nutritional status (CONUT), geriatric nutritional risk index (GNRI), and total cholesterol. Outcome definitions were heterogeneous across studies: some reported broader postoperative pulmonary complications (PPCs), whereas others focused specifically on postoperative pneumonia or postoperative pulmonary infection. In addition, biomarkers were modeled inconsistently across studies, including continuous, dichotomized, and ordinal-category approaches. Key study characteristics are summarized in Table 1, and detailed extracted data are presented in Supplementary Table S1A, S1B.

**Table 1:** Main study characteristics of the included studies.

Authors	Year	Country	Study design	Cancer type	Population/surgical cohort	Total (n)	Pulmonary events, *n* (%)	Preoperative marker(s) of interest	Outcome reported	Main adjusted extractable association(s)
Chen et al. (10)	2018	China	Single-center retrospective cohort	GC; stage NR	Elderly gastric cancer patients undergoing elective laparoscopic gastrectomy	262	35 PPCs (13.4%)	Albumin (also Hb and CRP screened)	PPCs within 30 days	Adjusted OR with 95% CI
Dai et al. (11)	2022	China	Single-center retrospective cohort	CRC; stage NR	Elderly patients receiving elective colorectal cancer surgery	638	38 PPCs (5.96%)	RDW; SII (also NLR and PLR screened)	PPCs	Adjusted ORs with 95% CIs
Han et al. (12)	2025	China	Single-center retrospective study	CRC; stage NR	Adults undergoing radical colorectal cancer surgery	866	72 POI (8.31%)	SIRI; AGR (also GNRI, PNI, NLR, PLR, SII, FPR, FAR screened)	Postoperative pulmonary infection (POI) during hospitalization	Adjusted ORs with 95% CIs
Inokuchi et al. (13)	2014	Japan	Single-center retrospective cohort	Gastric adenocarcinoma	Patients with gastric adenocarcinoma undergoing radical gastrectomy with lymphadenectomy	1,053	49 PPCs (4.7%)	Albumin (plus predicted VC as pulmonary variable)	PPCs and postoperative pneumonia	Adjusted ORs with 95% CIs for PPCs/pneumonia; biomarker only univariable
Kanno et al. (14)	2024	Japan	Single-center retrospective cohort	GC; stage/N stage reported	Patients undergoing gastrectomy for gastric cancer	108	26 infectious complications; pneumonia count NR in accessible text	Serum cholinesterase	Postoperative pneumonia (also infectious complications overall)	Adjusted logistic regression (*p* values in accessible text)
Kiuchi et al. (15)	2016	Japan	Single-center retrospective cohort	GC; pStage analyzed	Consecutive patients undergoing curative gastrectomy for gastric cancer	1,415	31 postoperative pneumonia (2.2%)	Albumin/nutritional status	Postoperative pneumonia	Adjusted ORs (95% CI partly not reported in abstract)
Li et al. (7)	2025	China	Single-center retrospective cohort	CRC; distant metastasis included as covariate	Patients undergoing radical surgery for colorectal cancer	2,553	230 PPCs (9.0%)	CONUT score	PPCs and postoperative pneumonia	Adjusted ORs with 95% CIs
Ma et al. (16)	2024	China	Single-center retrospective cohort	GC; pTNM and pT/pN analyzed	Patients undergoing D2 radical gastrectomy for gastric cancer	404	NR in accessible text	PNI; PLR (also CONUT and NLR assessed)	Postoperative pulmonary infection (POI) within 30 days	Multivariable logistic regression + nomogram
Mori et al. (5)	2021	Japan	Single-center retrospective cohort	Stage I-III GC	Stage I-III gastric cancer patients after curative gastrectomy	300	Pneumonia count not isolated in accessible text; infectious complications 54/300	NLR (systemic inflammatory prognostic parameters screened)	Postoperative pneumonia	Adjusted OR with 95% CI
Shoka et al. (6)	2020	Japan	Multicenter retrospective cohort	GC; resected cohort	Patients who underwent gastrectomy for gastric cancer at 9 institutions	1,415	42 grade II or higher postoperative pneumonia (3.0%)	Systemic inflammation score (SIS)	Postoperative pneumonia	Adjusted OR with 95% CI; ROC
Sun et al. (8)	2025	China	Single-center retrospective cohort	CRC; TNM stage recorded	Elderly colorectal cancer patients undergoing radical resection	339	40 PPI (11.8%)	CONUT; GNRI; PNI	Postoperative pulmonary infection (PPI)	Adjusted ORs with 95% CIs
Wu et al. (17)	2026	China	Two-center retrospective cohort	CRC	Elderly patients undergoing colorectal cancer resection	2,500	171 POP (6.8%)	Systemic immune-inflammation index (SII)	Postoperative pneumonia (POP)	Adjusted OR with 95% CI
Xiang et al. (18)	2025	China	Single-center retrospective cohort	CRC	Patients who underwent radical colorectal cancer resection	7,130	NR in accessible text	Albumin	Postoperative pneumonia	Adjusted OR with 95% CI + nomogram
Zhang et al. (19)	2015	China	Single-center retrospective cohort	GC	Gastric cancer patients with preoperative pulmonary function test undergoing gastrectomy	685 (derived from 124 PPCs, 18.1%)	124 PPCs (18.1%)	Albumin (plus Hb and pulmonary function)	PPCs	Adjusted association reported (exact OR/95% CI not visible in accessible text)
Zhou et al. (20)	2023	China	Single-center retrospective cohort	GC	Gastric cancer patients undergoing elective gastrectomy	2,124	150 PPCs (7.1%)	Total cholesterol (also albumin, CONUT, GNRI, PNI assessed)	PPCs	Adjusted ORs with 95% CIs + nomogram

AGR, albumin-to-globulin ratio; CONUT, controlling nutritional status; CRC, colorectal cancer; GC, gastric cancer; GNRI, geriatric nutritional risk index; NLR, neutrophil-to-lymphocyte ratio; PNI, prognostic nutritional index; PPCs, postoperative pulmonary complications; POI/PPI/POP, postoperative pulmonary infection/pneumonia; PLR, platelet-to-lymphocyte ratio; RDW, red blood cell distribution width; SII, systemic immune-inflammation index; SIRI, systemic inflammation response index; SIS, systemic inflammation score.

Methodological quality was generally moderate to high. All included studies received eight or nine stars on the Newcastle–Ottawa Scale, indicating an overall acceptable risk-of-bias profile for the included observational evidence (Table 2). Item-level NOS assessments are provided in Supplementary Table S2 to improve transparency and reproducibility.

**Table 2:** The Newcastle–Ottawa scale (NOS) assessment of the included studies.

Study	Year	Country	Type of article	The Newcastle–Ottawa Scale (NOS)		
				Selection	Comparability	Outcome
Chen et al. (10)	2018	China	Retrospective cohort	****	**	***
Dai et al. (11)	2022	China	Retrospective cohort	****	**	***
Han et al. (12)	2025	China	Retrospective cohort	****	**	***
Inokuchi et al. (13)	2014	Japan	Retrospective cohort	****	**	***
Kanno et al. (14)	2024	Japan	Retrospective cohort	****	*	***
Kiuchi et al. (15)	2016	Japan	Retrospective cohort	****	**	***
Li et al. (7)	2025	China	Retrospective cohort	****	**	***
Ma et al. (16)	2024	China	Retrospective cohort	****	**	***
Mori et al. (5)	2021	Japan	Retrospective cohort	****	**	***
Shoka et al. (6)	2020	Japan	Retrospective multicenter cohort	****	**	***
Sun et al. (8)	2025	China	Retrospective cohort	****	**	***
Wu et al. (17)	2026	China	Retrospective multicenter cohort	****	**	***
Xiang et al. (18)	2025	China	Retrospective cohort	****	**	***
Zhang et al. (19)	2015	China	Retrospective cohort	****	**	***
Zhou et al. (20)	2023	China	Retrospective cohort	****	**	***

* = 1 point; maximum score = points.

NOS was applied using the cohort-study domains of Selection, Comparability, and Outcome. Scores were assigned based on cohort representativeness, ascertainment of preoperative exposure, multivariable adjustment for key confounders, objective pulmonary-outcome assessment, and adequacy of postoperative follow-up.

### Narrative synthesis of biomarkers associated with PPCs

Evidence for broader PPCs was available mainly from gastric and colorectal surgery cohorts. In elderly patients undergoing laparoscopic gastrectomy for gastric cancer, PPCs occurred in 35 of 262 patients (13.4%), and preoperative albumin was identified as the only independent predictor in multivariable analysis (OR 1.15, 95% CI 1.06–1.28) (10). In elderly colorectal cancer surgery patients, PPCs occurred in 38 of 638 cases (5.96%), and preoperative RDW (OR 1.159, 95% CI 1.025–1.309) and preoperative SII (OR 1.001, 95% CI 1.000–1.003) were independently associated with PPCs (11). In a larger colorectal cancer cohort, PPCs occurred in 230 of 2,553 patients (9.0%), and higher CONUT category showed a graded association with increasing PPC risk, with ORs of 1.61 (95% CI 1.18–2.20) for mild malnutrition and 2.41 (95% CI 1.51–3.84) for moderate-to-severe malnutrition relative to normal nutritional status (7). Earlier gastric cancer evidence also linked PPCs to adverse survival outcomes, further underscoring the clinical relevance of pulmonary morbidity in this setting (19). Another gastric cancer study involving 2,124 patients reported PPCs in 150 cases (7.1%) and identified age >65 years, total cholesterol, total gastrectomy, longer operative duration, and oxycodone dose as independent predictors incorporated into a nomogram (20). By contrast, although lower preoperative albumin was associated with PPCs on univariable analysis in a radical gastrectomy cohort, the final multivariable model retained extended operating time, total gastrectomy, and predicted vital capacity rather than nutritional biomarkers (13).

Taken together, studies focused on broader PPCs suggest that both inflammatory and nutritional variables may contribute to postoperative pulmonary morbidity after gastrointestinal cancer surgery. However, the evidence remained fragmented because the biomarkers examined differed substantially across studies, and even conceptually similar markers were analyzed on different statistical scales.

### Narrative synthesis of biomarkers associated with postoperative pneumonia or postoperative pulmonary infection

#### Albumin-related findings

Albumin-related findings were reported in both gastric and colorectal surgery cohorts. In gastric cancer, postoperative pneumonia occurred in 31 of 1,413 patients (2.2%), and low albumin (<3.0 g/mL) was independently associated with postoperative pneumonia (15). In elderly colorectal cancer patients, postoperative pneumonia occurred in 171 of 2,500 cases (6.8%), and albumin <35 g/L was likewise retained as an independent predictor in multivariable analysis (17). A large colorectal cancer nomogram study also identified preoperative albumin as one of the independent predictors of postoperative pneumonia, although a biomarker-specific adjusted OR was not emphasized in the main report (18). By contrast, in a smaller gastric cancer cohort, albumin was associated with postoperative pneumonia on univariable analysis but was not retained in the final multivariable pneumonia model after adjustment for other clinicopathological variables (14).

Overall, the direction of association was broadly consistent, with lower preoperative albumin indicating a higher risk of postoperative infectious pulmonary events.

#### Cholinesterase

The clearest evidence for cholinesterase came from a gastric cancer cohort in which 26 of 108 patients (24%) developed postoperative infectious complications. Postoperative pneumonia occurred significantly more often in the low-cholinesterase group, and cholinesterase <219 U/L remained independently associated with postoperative pneumonia in multivariable analysis, together with age, cerebrovascular comorbidities, and total gastrectomy (14).

#### NLR

In 300 patients with stage I–III gastric cancer undergoing curative gastrectomy, 101 (33.7%) developed Clavien–Dindo grade II–IV postoperative complications and 54 (18.0%) developed infectious complications. Among the evaluated systemic inflammatory indices, postoperative pneumonia appeared particularly sensitive to these markers, and preoperative NLR emerged as an independent predictor of postoperative pneumonia in multivariable analysis (OR 14.621, 95% CI 1.160–184.348) (5).

#### SIS

In a large multi-institutional gastric cancer cohort, grade II or higher postoperative complications occurred in 319 patients (22.5%), and postoperative pneumonia occurred in 42 patients (3.0%). Preoperative SIS yielded the highest AUC among 31 candidate variables for predicting postoperative pneumonia, with an optimal cutoff of 2, and SIS ≥2 remained independently associated with postoperative pneumonia in multivariable analysis (OR 2.31, 95% CI 1.19–4.48) (6).

#### SII

SII was evaluated in both PPC- and pneumonia-focused studies, but the clearest infectious pulmonary endpoint came from a multicenter colorectal cancer cohort in which postoperative pneumonia occurred in 171 of 2,500 patients (6.8%). In that study, the highest quartile of preoperative SII was independently associated with postoperative pneumonia (OR 6.017, 95% CI 3.377–10.72) (17). In a separate elderly colorectal surgery cohort, preoperative SII was also independently associated with broader PPCs, further supporting the clinical relevance of this marker (11).

#### PNI, CONUT, and GNRI

Evidence for nutrition-based composite indices was strongest in colorectal cancer cohorts. In elderly patients undergoing radical colorectal cancer surgery, 40 of 339 patients (11.8%) developed postoperative pulmonary infection, and multivariable analysis identified elevated CONUT (OR 2.23, 95% CI 1.25–3.98), decreased GNRI (OR 0.94, 95% CI 0.90–0.99), and decreased PNI (OR 0.88, 95% CI 0.81–0.97) as independent predictors (8). In another colorectal cancer cohort, higher CONUT category was associated not only with broader PPCs but also with postoperative pneumonia as a secondary outcome, again indicating an unfavorable gradient with worsening nutritional status (7).

#### PNI and PLR in gastric cancer surgery

In a gastric cancer study focused on postoperative pulmonary infection after D2 radical gastrectomy, both PNI and PLR were retained in the final multivariable model. Higher PNI (>48.55) was associated with a lower risk of postoperative pulmonary infection (OR 0.529, 95% CI 0.313–0.893), and higher PLR (>142.89) was likewise associated with lower risk (OR 0.489, 95% CI 0.290–0.822) (16). Although the direction reported for PLR was somewhat counterintuitive relative to conventional inflammatory-risk expectations, the findings still suggest that preoperative composite nutritional and hematologic indices may contribute to pulmonary infection risk stratification after gastric cancer surgery.

#### SIRI and AGR

In a colorectal cancer cohort of 866 patients undergoing radical surgery, independent predictors of postoperative pulmonary infection included age, respiratory disease, SIRI, AGR, operative method, and operative duration (12). Although the exact biomarker-specific ORs for SIRI and AGR were not fully reported in the summary data available here, both markers were retained in the final multivariable model and contributed to the predictive nomogram, supporting their potential relevance in postoperative pulmonary infection risk assessment.

### Prognostic implications of postoperative pneumonia

A small number of studies also addressed the prognostic implications of postoperative infectious pulmonary events beyond immediate morbidity. In gastric cancer, postoperative pneumonia was associated with significantly poorer cancer-related survival and remained an independent prognostic factor in multivariable Cox analysis (HR 2.32, 95% CI 1.17–4.15) (15). In elderly colorectal cancer patients, postoperative pneumonia was likewise associated with worse short-term complication profiles and a markedly higher 2-year mortality rate (17). These findings suggest that infectious pulmonary complications after gastrointestinal cancer surgery may reflect not only perioperative vulnerability but also broader adverse postoperative trajectories.

## Discussion

In this systematic review and narrative synthesis, the available evidence consistently suggested that adverse preoperative inflammatory and immune-nutritional profiles were associated with a higher risk of postoperative pulmonary morbidity after gastrointestinal cancer surgery. Although the included studies varied substantially in design, endpoint definition, and analytic strategy, a recurrent pattern emerged across both gastric and colorectal cancer cohorts: markers reflecting heightened systemic inflammation, such as NLR, SIS, and SII, were associated with increased risk of postoperative pneumonia or broader PPCs, while markers reflecting impaired nutritional reserve, including albumin, CONUT, PNI, and GNRI, likewise indicated greater pulmonary vulnerability. Taken together, these findings suggest that postoperative pulmonary risk in this setting may be interpreted as being associated with a preoperative host state characterized by combined inflammatory burden and nutritional impairment, rather than the isolated effect of any single biomarker. However, these blood-based markers should not be interpreted as direct evidence that tumour-derived inflammation alone causes postoperative pulmonary complications. They are non-specific indicators that may also reflect age, comorbidities, frailty, organ dysfunction, tumour burden, and overall perioperative physiological reserve. Even after surgical removal of the primary tumour, this preoperative vulnerability may persist into the early postoperative period, when anaesthesia, operative stress, pain, immobility, impaired cough, and postoperative inflammatory activation may act as additional triggers for pulmonary infection or respiratory deterioration. This interpretation is clinically relevant because it places routinely available laboratory indices within a broader perioperative risk phenotype that may influence susceptibility to infection, respiratory decompensation, delayed recovery, and early adverse postoperative trajectories.

These associations are biologically plausible in the context of perioperative host vulnerability. The inflammatory markers highlighted in the included studies are unlikely to function merely as numerical predictors; rather, they may reflect a preoperative state of dysregulated host defence that predisposes patients to pulmonary infection and respiratory deterioration after major gastrointestinal cancer surgery. Elevated NLR, SII, and SIS generally capture a pattern of neutrophilia, platelet activation, and relative lymphopenia, suggesting intensified innate inflammatory signalling alongside impaired adaptive immune competence (21, 22). Under perioperative stress, such an imbalance may increase pulmonary vulnerability by facilitating postoperative infection, amplifying local and systemic inflammatory responses, and impairing resolution of tissue injury after anaesthesia and surgery (21). This interpretation is supported by the study-level findings of the present review. In gastric cancer, preoperative NLR was independently associated with postoperative pneumonia, with an OR of 14.621 (95% CI 1.160–184.348) (5), whereas in elderly colorectal cancer patients the highest quartile of preoperative SII was independently associated with postoperative pneumonia (OR 6.017, 95% CI 3.377–10.72) (17). The repeated association of inflammatory indices with both postoperative pneumonia and broader PPCs across gastric and colorectal surgery cohorts therefore likely reflects a clinically meaningful inflammatory phenotype rather than an incidental statistical pattern. In addition, the bidirectional relationship described by Wu et al. suggests that baseline inflammatory burden and postoperative pulmonary infection may further amplify one another once complications occur, pointing to a self-reinforcing perioperative inflammatory cycle rather than two isolated events (17).

A similar interpretation applies to the nutritional markers identified in the included studies. Low albumin, low PNI, low GNRI, and high CONUT are better understood not simply as isolated laboratory abnormalities, but as indicators of reduced physiological reserve, impaired immune-nutritional status, and limited capacity to withstand the catabolic stress of major cancer surgery (4, 23–26). From a pulmonary perspective, this may translate into weaker anti-infective defence, less effective tissue repair, poorer recovery of respiratory muscle function, and less efficient coughing and secretion clearance after surgery (4, 24, 25). The current review again provides study-level support for this interpretation. In gastric cancer, low preoperative albumin (<3.0 g/mL) was independently associated with postoperative pneumonia (15), while in elderly colorectal cancer patients poorer nutritional status, reflected by higher CONUT and lower GNRI and PNI, was independently associated with postoperative pulmonary infection (8). Importantly, inflammatory activation and nutritional depletion often coexist in patients undergoing gastrointestinal cancer surgery, particularly in older adults, in those with more advanced disease, and in those exposed to substantial operative stress. For that reason, the present findings are more plausibly interpreted as different biomarker expressions of the same adverse host condition than as unrelated signals acting independently. This view is also consistent with contemporary perioperative nutrition guidance, which emphasizes systematic nutritional risk assessment in major cancer surgery as part of broader perioperative optimization (4). Taken together, these mechanistic considerations help explain why the observed biomarker associations may still carry clinical value even in the absence of a single dominant marker.

From an integrative perspective, the most clinically relevant signals in the current evidence base appear to come from commonly available markers with relatively recurrent support, particularly albumin, CONUT, PNI, NLR, SIS, and SII. Albumin was commonly evaluated through low-albumin categories, while representative study-specific thresholds or categories for other markers included CONUT-defined mild or moderate-to-severe malnutrition, SIS ≥2, PNI >48.55 in one gastric cancer cohort, and higher-category or quartile-based SII. For markers such as NLR and SII, thresholds or analytic categories varied across studies, so no universal cutoff can currently be recommended. These thresholds should therefore be interpreted as study-specific reference points rather than clinically established decision thresholds.

From a clinical standpoint, the present findings do not support the routine implementation of any single biomarker as a stand-alone decision-making tool, but they do support the practical value of recognising an adverse inflammatory–nutritional profile as an adjunct to perioperative risk assessment. For surgeons, anaesthesiologists, and perioperative teams, markers such as low albumin, high inflammatory indices, or composite nutritional impairment may be most useful when interpreted as adjuncts to overall clinical assessment rather than as isolated thresholds. In practical terms, these abnormalities may help identify patients who warrant closer pulmonary surveillance, more careful perioperative optimisation, and heightened attention to postoperative respiratory complications. They may also help identify patients in whom supportive strategies such as nutritional optimisation, structured pulmonary prehabilitation, and intensified postoperative respiratory care deserve greater consideration, although the effectiveness of such measures still depends on how they are applied and in whom they are used (4, 27–30). Importantly, recent perioperative evidence suggests that risk identification alone is not sufficient; the clinical value of biomarker-based stratification ultimately depends on whether it can be linked to interventions that are feasible, targeted, and capable of improving outcomes in higher-risk patients (29, 30).

At the same time, the current literature reveals an important tension in the field. Association studies and prediction-model studies answer different questions and should not be interpreted interchangeably. A notable feature of several included studies is the rapid shift from identifying biomarker associations to building nomograms or multivariable prediction models, often accompanied by internal ROC/AUC analysis, calibration plots, and decision-curve analysis (8, 12, 16, 18, 20). These studies are informative and should not be dismissed; however, their translation into routine practice remains limited. A statistically well-performing model is not necessarily a clinically superior one, particularly when external validation is lacking, biological interpretability is weak, or the added value of model complexity over simpler markers such as albumin or NLR remains uncertain (3, 28, 31). In the present evidence base, some models reported apparently acceptable internal discrimination—for example, the Ma nomogram reported a C-index of 0.707, the Han study reported an AUC of 0.773, and the Xiang nomogram reported AUCs of 0.745 and 0.773 in the training and validation cohorts, respectively (12, 16, 18). Yet these performance metrics alone do not establish generalisability, transportability, or meaningful clinical gain. More broadly, contemporary guidance on prediction model development and appraisal has emphasized that discrimination should not be interpreted in isolation and that clinical usefulness depends on calibration, validation, and incremental value in real practice settings (28, 31). For this reason, the current model-building literature should be viewed as hypothesis-generating and clinically suggestive rather than definitive. What the field needs next is not simply more model construction, but rigorous validation of whether a smaller set of biologically interpretable and easily obtainable markers can improve perioperative decision-making beyond standard clinical assessment.

In colorectal cancer surgery, the current evidence shows a convergent but heterogeneous signal across inflammatory and nutritional domains. In the included studies, poorer preoperative nutritional status measured by CONUT, PNI, GNRI, or albumin was associated with postoperative pulmonary complications, postoperative pulmonary infection, or postoperative pneumonia, while inflammatory markers such as SII and SIRI were also retained in several colorectal cancer cohorts or prediction models (7, 8, 11, 12, 17, 18). In addition, Evirgen and Çetin recently evaluated postoperative day-7 C-reactive protein-to-albumin ratio (CAR) in patients undergoing colorectal cancer surgery and reported that higher postoperative CAR was associated with 30-day postoperative complications (32). Although that study differs from the present review because it assessed a postoperative biomarker and overall surgical complications rather than preoperative markers and pulmonary outcomes, it provides relevant contextual evidence. Together, these findings suggest that inflammation–nutrition-based biomarkers may have perioperative value in colorectal cancer surgery, but their interpretation depends strongly on biomarker timing, outcome definition, and clinical context.

Within the constraints of the current literature, this study has several strengths. First, it addressed a focused and clinically relevant question in a field where postoperative pulmonary risk remains important but biomarker-based evidence has been scattered across different tumour sites, pulmonary endpoints, and analytic approaches. By restricting the review to gastrointestinal cancer surgery and prespecifying PPCs as the primary outcome framework, with postoperative pneumonia and other individual pulmonary complications extracted separately, we reduced conceptual ambiguity while preserving clinical relevance. Second, the review was prospectively registered and conducted in accordance with PRISMA 2020 guidance, with duplicate screening, data extraction, and structured methodological quality assessment. Third, the synthesis strategy was deliberately aligned with the structure of the available evidence. Rather than forcing a pooled estimate across clearly dissimilar biomarkers, scales, and pulmonary endpoints, we preserved biomarker-specific and outcome-specific distinctions and prioritized clinical interpretability over statistical convenience. This approach was intended to transparently manage heterogeneous evidence rather than overcome it, particularly because many associations were supported by single studies and formal pooling could have obscured clinically meaningful differences. Finally, by integrating both broader PPC outcomes and infection-focused pulmonary endpoints, the review provides a more complete picture of how preoperative inflammatory and nutritional imbalance may influence perioperative pulmonary risk after gastrointestinal cancer surgery.

Several limitations should be acknowledged. First, the evidence base consisted exclusively of observational studies, most of them retrospective and single-center in design, which increases susceptibility to selection bias, residual confounding, and instability of effect estimates. Because patient selection, perioperative management, pulmonary complication surveillance, and covariate adjustment may have differed across centers and studies, the observed associations should be interpreted as exploratory rather than definitive. Second, substantial heterogeneity was present across studies in tumour site, surgical population, pulmonary endpoint definition, biomarker choice, cutoff derivation, and modeling strategy. This heterogeneity limited direct comparability between studies and reduced confidence in the reproducibility of any single biomarker signal. Some studies evaluated composite PPCs, whereas others focused specifically on postoperative pneumonia or postoperative pulmonary infection; similarly, biomarkers were variably analyzed as continuous variables, dichotomized cutoffs, or ordinal categories. Third, although multivariable-adjusted results were preferentially interpreted, adjustment strategies were not uniform, and not all studies provided adjusted estimates for all biomarkers of interest. Unmeasured or inconsistently adjusted factors, such as baseline pulmonary function, smoking status, comorbidities, surgical extent, anesthesia management, and perioperative respiratory care, may therefore have influenced the reported associations. Fourth, evidence strength varied by biomarker: albumin, NLR, SIS, SII, PNI, and CONUT had relatively recurrent support, whereas several other markers had only limited single-study evidence. Fifth, several included studies were primarily nomogram-development analyses rather than biomarker-validation studies. These designs are clinically informative, but they may conflate predictive utility with biological or causal interpretation, particularly when internally derived models are emphasized without external validation. Finally, the current literature remains insufficient to determine whether more complex multivariable models provide meaningful clinical benefit over simpler, readily available inflammatory or nutritional markers in routine perioperative practice.

In conclusion, the current evidence indicates that postoperative pulmonary risk after gastric and colorectal cancer surgery is closely linked to a combined preoperative state of systemic inflammation and impaired nutritional reserve. These biomarkers should therefore be viewed not as isolated laboratory abnormalities, but as clinically accessible indicators of an adverse host condition that may complement conventional perioperative risk assessment. However, because the available evidence remains heterogeneous in pulmonary outcome definitions, biomarker modeling, cutoff selection, and validation strategy, these markers should not be used as stand-alone decision-making tools or interpreted as having established universal thresholds. Until such evidence becomes available, inflammatory and immune-nutritional biomarkers are best used to enrich clinical judgment rather than to replace it.

## Data Availability

The data analyzed in this study is subject to the following licenses/restrictions: All data generated or analyzed during this study are included in this published article and its supplementary information files. Additional details are available from the first author upon reasonable request. Requests to access these datasets should be directed to 17744605071@sina.cn.
