# Supplementary material for: Preoperative inflammatory and immune-nutritional markers and postoperative pulmonary complications after gastric and colorectal cancer surgery: a systematic review and narrative synthesis
Source: Front Surg. 2026 Jul 2;13:1850606. doi: 10.3389/fsurg.2026.1850606 (PMC13372747; doi:10.3389/fsurg.2026.1850606)
Supplement: Supplementary file 1 [file Supplementaryfile1.zip › Supplementary methods.DOCX]

**Supplementary Methods: Literature search strategy**

We systematically searched PubMed, Embase, Web of Science Core Collection, Scopus, and the Cochrane Library to identify studies evaluating the association of preoperative inflammatory and immune-nutritional markers with postoperative pulmonary complications after gastrointestinal cancer surgery. The searches were performed from database inception to 3 March 2026. In addition, the reference lists of all included studies and relevant reviews were manually screened to identify any further eligible studies. The detailed search strategies for each database are provided below.

**1. PubMed**

**Database:** PubMed (MEDLINE)

**Date range:** from database inception to 3 March 2026

**Search field:** Title/Abstract and related indexing terms

**Search strategy:**

((("gastrointestinal cancer*" OR "gastrointestinal neoplasm*" OR "digestive system neoplasm*" OR "digestive tract cancer*" OR "digestive tract neoplasm*" OR "gastric cancer" OR "stomach cancer" OR "gastric neoplasm*" OR "gastric carcinoma*" OR "gastroesophageal junction adenocarcinoma" OR "esophagogastric junction cancer" OR "colorectal cancer" OR "colorectal neoplasm*" OR "colon cancer" OR "colonic neoplasm*" OR "rectal cancer" OR "rectal neoplasm*" OR "bowel cancer"))

AND

("gastrointestinal surgery" OR "gastrointestinal cancer surgery" OR "digestive surgery" OR gastrectom* OR "gastric surgery" OR colectom* OR proctectom* OR proctocolectom* OR "colorectal surgery" OR "colon surgery" OR "rectal surgery" OR "bowel resection" OR "intestinal resection" OR "cancer surgery")

AND

(preoperativ* OR presurg* OR "before surgery" OR "before operation")

AND

("inflammation-based biomarker*" OR "inflammatory biomarker*" OR "inflammatory marker*" OR "immune-nutritional marker*" OR "immunonutritional marker*" OR "nutritional marker*" OR "inflammation-based index" OR "systemic inflammatory index" OR "systemic immune-inflammation index" OR SII OR NLR OR "neutrophil-to-lymphocyte ratio" OR PLR OR "platelet-to-lymphocyte ratio" OR LMR OR "lymphocyte-to-monocyte ratio" OR SIRI OR "systemic inflammation response index" OR PIV OR "pan-immune inflammation value" OR PNI OR "prognostic nutritional index" OR AISI OR "aggregate index of systemic inflammation" OR CONUT OR "controlling nutritional status" OR GNRI OR "geriatric nutritional risk index" OR SIS OR "systemic inflammation score" OR WBC OR "white blood cell*" OR neutrophil* OR lymphocyte* OR monocyte* OR RDW OR "red cell distribution width" OR CRP OR "C-reactive protein" OR albumin OR prealbumin OR "C-reactive protein-to-albumin ratio" OR CAR)

AND

("postoperative pulmonary complication*" OR PPC OR PPCs OR "postoperative pulmonary morbidity" OR "pulmonary complication*" OR "postoperative pneumonia" OR "postoperative pulmonary infection" OR "post-surgical pneumonia" OR "postoperative lung infection" OR "pulmonary infection" OR atelectasis OR "respiratory failure" OR reintubation OR "prolonged mechanical ventilation" OR "postoperative ventilatory support" OR ARDS OR "acute respiratory distress syndrome"))

NOT

(review OR "systematic review" OR meta-analysis OR metaanalysis OR editorial OR letter OR "case report" OR "case reports" OR "conference abstract" OR "meeting abstract" OR "oral presentation" OR poster)

**2. Embase**

**Database:** Embase

**Date range:** from database inception to 3 March 2026

**Search strategy:**

('gastrointestinal cancer*' OR 'gastrointestinal neoplasm*' OR 'digestive system neoplasm*' OR 'digestive tract cancer*' OR 'digestive tract neoplasm*' OR 'gastric cancer' OR 'stomach cancer' OR 'gastric neoplasm*' OR 'gastric carcinoma*' OR 'gastroesophageal junction adenocarcinoma' OR 'esophagogastric junction cancer' OR 'colorectal cancer' OR 'colorectal neoplasm*' OR 'colon cancer' OR 'colonic neoplasm*' OR 'rectal cancer' OR 'rectal neoplasm*' OR 'bowel cancer')

AND

('gastrointestinal surgery' OR 'gastrointestinal cancer surgery' OR 'digestive surgery' OR gastrectom* OR 'gastric surgery' OR colectom* OR proctectom* OR proctocolectom* OR 'colorectal surgery' OR 'colon surgery' OR 'rectal surgery' OR 'bowel resection' OR 'intestinal resection' OR 'cancer surgery')

AND

(preoperativ* OR presurg* OR 'before surgery' OR 'before operation')

AND

('inflammation-based biomarker*' OR 'inflammatory biomarker*' OR 'inflammatory marker*' OR 'immune-nutritional marker*' OR 'immunonutritional marker*' OR 'nutritional marker*' OR 'inflammation-based index' OR 'systemic inflammatory index' OR 'systemic immune-inflammation index' OR SII OR NLR OR 'neutrophil-to-lymphocyte ratio' OR PLR OR 'platelet-to-lymphocyte ratio' OR LMR OR 'lymphocyte-to-monocyte ratio' OR SIRI OR 'systemic inflammation response index' OR PIV OR 'pan-immune inflammation value' OR PNI OR 'prognostic nutritional index' OR AISI OR 'aggregate index of systemic inflammation' OR CONUT OR 'controlling nutritional status' OR GNRI OR 'geriatric nutritional risk index' OR SIS OR 'systemic inflammation score' OR WBC OR 'white blood cell*' OR neutrophil* OR lymphocyte* OR monocyte* OR RDW OR 'red cell distribution width' OR CRP OR 'C-reactive protein' OR albumin OR prealbumin OR 'C-reactive protein-to-albumin ratio' OR CAR)

AND

('postoperative pulmonary complication*' OR PPC OR PPCs OR 'postoperative pulmonary morbidity' OR 'pulmonary complication*' OR 'postoperative pneumonia' OR 'postoperative pulmonary infection' OR 'post-surgical pneumonia' OR 'postoperative lung infection' OR 'pulmonary infection' OR atelectasis OR 'respiratory failure' OR reintubation OR 'prolonged mechanical ventilation' OR 'postoperative ventilatory support' OR ARDS OR 'acute respiratory distress syndrome')

NOT

('review' OR 'systematic review' OR 'meta analysis' OR 'editorial' OR 'letter' OR 'case report' OR 'conference abstract' OR 'meeting abstract' OR 'oral presentation' OR poster)

**3. Web of Science Core Collection**

**Database:** Web of Science Core Collection

**Date range:** from database inception to 3 March 2026

**Search field:** Topic (TS)

**Search strategy:**

TS=('gastrointestinal cancer*' OR 'gastrointestinal neoplasm*' OR 'digestive system neoplasm*' OR 'digestive tract cancer*' OR 'digestive tract neoplasm*' OR 'gastric cancer' OR 'stomach cancer' OR 'gastric neoplasm*' OR 'gastric carcinoma*' OR 'gastroesophageal junction adenocarcinoma' OR 'esophagogastric junction cancer' OR 'colorectal cancer' OR 'colorectal neoplasm*' OR 'colon cancer' OR 'colonic neoplasm*' OR 'rectal cancer' OR 'rectal neoplasm*' OR 'bowel cancer')

AND

('gastrointestinal surgery' OR 'gastrointestinal cancer surgery' OR 'digestive surgery' OR gastrectom* OR 'gastric surgery' OR colectom* OR proctectom* OR proctocolectom* OR 'colorectal surgery' OR 'colon surgery' OR 'rectal surgery' OR 'bowel resection' OR 'intestinal resection' OR 'cancer surgery')

AND

(preoperativ* OR presurg* OR 'before surgery' OR 'before operation')

AND

('inflammation-based biomarker*' OR 'inflammatory biomarker*' OR 'inflammatory marker*' OR 'immune-nutritional marker*' OR 'immunonutritional marker*' OR 'nutritional marker*' OR 'inflammation-based index' OR 'systemic inflammatory index' OR 'systemic immune-inflammation index' OR SII OR NLR OR 'neutrophil-to-lymphocyte ratio' OR PLR OR 'platelet-to-lymphocyte ratio' OR LMR OR 'lymphocyte-to-monocyte ratio' OR SIRI OR 'systemic inflammation response index' OR PIV OR 'pan-immune inflammation value' OR PNI OR 'prognostic nutritional index' OR AISI OR 'aggregate index of systemic inflammation' OR CONUT OR 'controlling nutritional status' OR GNRI OR 'geriatric nutritional risk index' OR SIS OR 'systemic inflammation score' OR WBC OR 'white blood cell*' OR neutrophil* OR lymphocyte* OR monocyte* OR RDW OR 'red cell distribution width' OR CRP OR 'C-reactive protein' OR albumin OR prealbumin OR 'C-reactive protein-to-albumin ratio' OR CAR)

AND

('postoperative pulmonary complication*' OR PPC OR PPCs OR 'postoperative pulmonary morbidity' OR 'pulmonary complication*' OR 'postoperative pneumonia' OR 'postoperative pulmonary infection' OR 'post-surgical pneumonia' OR 'postoperative lung infection' OR 'pulmonary infection' OR atelectasis OR 'respiratory failure' OR reintubation OR 'prolonged mechanical ventilation' OR 'postoperative ventilatory support' OR ARDS OR 'acute respiratory distress syndrome')

NOT

('review' OR 'systematic review' OR 'meta analysis' OR 'editorial' OR 'letter' OR 'case report' OR 'conference abstract' OR 'meeting abstract' OR 'oral presentation' OR poster)

**4. Scopus**

**Database:** Scopus

**Date range:** from database inception to 3 March 2026

**Search field:** TITLE-ABS-KEY

**Search strategy:**

TITLE-ABS-KEY( ( "gastrointestinal cancer*" OR "gastrointestinal neoplasm*" OR "digestive system neoplasm*" OR "digestive tract cancer*" OR "digestive tract neoplasm*" OR "gastric cancer" OR "stomach cancer" OR "gastric neoplasm*" OR "gastric carcinoma*" OR "gastroesophageal junction adenocarcinoma" OR "esophagogastric junction cancer" OR "colorectal cancer" OR "colorectal neoplasm*" OR "colon cancer" OR "colonic neoplasm*" OR "rectal cancer" OR "rectal neoplasm*" OR "bowel cancer" )

AND

( "gastrointestinal surgery" OR "gastrointestinal cancer surgery" OR "digestive surgery" OR gastrectom* OR "gastric surgery" OR colectom* OR proctectom* OR proctocolectom* OR "colorectal surgery" OR "colon surgery" OR "rectal surgery" OR "bowel resection" OR "intestinal resection" OR "cancer surgery" )

AND

(preoperativ* OR presurg* OR "before surgery" OR "before operation")

AND

( SII OR NLR OR PLR OR LMR OR SIRI OR PIV OR PNI OR AISI OR CONUT OR GNRI OR SIS OR WBC OR RDW OR CRP OR albumin OR prealbumin OR CAR OR "systemic immune-inflammation index" OR "neutrophil-to-lymphocyte ratio" OR "platelet-to-lymphocyte ratio" OR "lymphocyte-to-monocyte ratio" OR "systemic inflammation response index" OR "pan-immune inflammation value" OR "prognostic nutritional index" OR "aggregate index of systemic inflammation" OR "controlling nutritional status" OR "geriatric nutritional risk index" OR "systemic inflammation score" OR "red cell distribution width" OR "C-reactive protein" OR neutrophil* OR lymphocyte* OR monocyte* )

AND

( "postoperative pulmonary complication*" OR PPC OR PPCs OR "pulmonary complication*" OR "postoperative pulmonary morbidity" OR "postoperative pneumonia" OR "postoperative pulmonary infection" OR "post-surgical pneumonia" OR "postoperative lung infection" OR "pulmonary infection" OR atelectasis OR "respiratory failure" OR reintubation OR "prolonged mechanical ventilation" OR "postoperative ventilatory support" OR ARDS OR "acute respiratory distress syndrome" ) )

AND NOT TITLE-ABS-KEY( review OR "systematic review" OR "meta analysis" OR editorial OR letter OR "case report" OR "conference abstract" OR "meeting abstract" OR "oral presentation" OR poster )

**5. Cochrane Library**

**Database:** Cochrane Library

**Date range:** from database inception to 3 March 2026

**Search strategy:**

(('gastrointestinal cancer*' OR 'gastrointestinal neoplasm*' OR 'digestive system neoplasm*' OR 'digestive tract cancer*' OR 'digestive tract neoplasm*' OR 'gastric cancer' OR 'stomach cancer' OR 'gastric neoplasm*' OR 'gastric carcinoma*' OR 'gastroesophageal junction adenocarcinoma' OR 'esophagogastric junction cancer' OR 'colorectal cancer' OR 'colorectal neoplasm*' OR 'colon cancer' OR 'colonic neoplasm*' OR 'rectal cancer' OR 'rectal neoplasm*' OR 'bowel cancer')

AND

('gastrointestinal surgery' OR 'gastrointestinal cancer surgery' OR 'digestive surgery' OR gastrectom* OR 'gastric surgery' OR colectom* OR proctectom* OR proctocolectom* OR 'colorectal surgery' OR 'colon surgery' OR 'rectal surgery' OR 'bowel resection' OR 'intestinal resection' OR 'cancer surgery')

AND

(preoperativ* OR presurg* OR 'before surgery' OR 'before operation')

AND

('inflammation-based biomarker*' OR 'inflammatory biomarker*' OR 'inflammatory marker*' OR 'immune-nutritional marker*' OR 'immunonutritional marker*' OR 'nutritional marker*' OR 'inflammation-based index' OR 'systemic inflammatory index' OR 'systemic immune-inflammation index' OR SII OR NLR OR 'neutrophil-to-lymphocyte ratio' OR PLR OR 'platelet-to-lymphocyte ratio' OR LMR OR 'lymphocyte-to-monocyte ratio' OR SIRI OR 'systemic inflammation response index' OR PIV OR 'pan-immune inflammation value' OR PNI OR 'prognostic nutritional index' OR AISI OR 'aggregate index of systemic inflammation' OR CONUT OR 'controlling nutritional status' OR GNRI OR 'geriatric nutritional risk index' OR SIS OR 'systemic inflammation score' OR WBC OR 'white blood cell*' OR neutrophil* OR lymphocyte* OR monocyte* OR RDW OR 'red cell distribution width' OR CRP OR 'C-reactive protein' OR albumin OR prealbumin OR 'C-reactive protein-to-albumin ratio' OR CAR)

AND

('postoperative pulmonary complication*' OR PPC OR PPCs OR 'postoperative pulmonary morbidity' OR 'pulmonary complication*' OR 'postoperative pneumonia' OR 'postoperative pulmonary infection' OR 'post-surgical pneumonia' OR 'postoperative lung infection' OR 'pulmonary infection' OR atelectasis OR 'respiratory failure' OR reintubation OR 'prolonged mechanical ventilation' OR 'postoperative ventilatory support' OR ARDS OR 'acute respiratory distress syndrome'))
