# Supplementary material for: Preoperative inflammatory and immune-nutritional markers and postoperative pulmonary complications after gastric and colorectal cancer surgery: a systematic review and narrative synthesis
Source: Front Surg. 2026 Jul 2;13:1850606. doi: 10.3389/fsurg.2026.1850606 (PMC13372747; doi:10.3389/fsurg.2026.1850606)
Supplement: Supplementary file 1 [file Supplementaryfile1.zip › Supplementary Table s2.DOCX]

| Supplementary Table S2. Item-level Newcastle–Ottawa Scale (NOS) assessment of the included studies | | | | | | | | | | | | | | | | |
| --- | --- | --- | --- | --- | --- | --- | --- | --- | --- | --- | --- | --- | --- | --- | --- | --- |
| Study | Year | Country | Type of article | S1 | S2 | S3 | S4 | Selection | C1 | C2 | Comparability | O1 | O2 | O3 | Outcome | Total NOS score |
| Chen et al. [10] | 2018 | China | Retrospective cohort | * | * | * | * | **** | * | * | ** | * | * | * | *** | 9 |
| Dai et al. [11] | 2022 | China | Retrospective cohort | * | * | * | * | **** | * | * | ** | * | * | * | *** | 9 |
| Han et al. [12] | 2025 | China | Retrospective cohort | * | * | * | * | **** | * | * | ** | * | * | * | *** | 9 |
| Inokuchi et al. [13] | 2014 | Japan | Retrospective cohort | * | * | * | * | **** | * | * | ** | * | * | * | *** | 9 |
| Kanno et al. [14] | 2024 | Japan | Retrospective cohort | * | * | * | * | **** | * |  | * | * | * | * | *** | 8 |
| Kiuchi et al. [15] | 2016 | Japan | Retrospective cohort | * | * | * | * | **** | * | * | ** | * | * | * | *** | 9 |
| Li et al. [7] | 2025 | China | Retrospective cohort | * | * | * | * | **** | * | * | ** | * | * | * | *** | 9 |
| Ma et al. [16] | 2024 | China | Retrospective cohort | * | * | * | * | **** | * | * | ** | * | * | * | *** | 9 |
| Mori et al. [5] | 2021 | Japan | Retrospective cohort | * | * | * | * | **** | * | * | ** | * | * | * | *** | 9 |
| Shoka et al. [6] | 2020 | Japan | Retrospective multicenter cohort | * | * | * | * | **** | * | * | ** | * | * | * | *** | 9 |
| Sun et al. [8] | 2025 | China | Retrospective cohort | * | * | * | * | **** | * | * | ** | * | * | * | *** | 9 |
| Wu et al. [17] | 2026 | China | Retrospective multicenter cohort | * | * | * | * | **** | * | * | ** | * | * | * | *** | 9 |
| Xiang et al. [18] | 2025 | China | Retrospective cohort | * | * | * | * | **** | * | * | ** | * | * | * | *** | 9 |
| Zhang et al. [19] | 2015 | China | Retrospective cohort | * | * | * | * | **** | * | * | ** | * | * | * | *** | 9 |
| Zhou et al. [20] | 2023 | China | Retrospective cohort | * | * | * | * | **** | * | * | ** | * | * | * | *** | 9 |
| Note: * = 1 point; blank = 0 point; maximum score = 9 points. NOS was applied using the cohort-study domains of Selection, Comparability, and Outcome. S1 = representativeness of exposed cohort; S2 = selection of non-exposed cohort; S3 = ascertainment of exposure; S4 = outcome not present at baseline. C1 = adjustment for key confounders; C2 = adjustment for additional confounders. O1 = assessment of outcome; O2 = follow-up long enough for outcome; O3 = adequacy of follow-up. | | | | | | | | | | | | | | | | |
