# Supplementary material for: Preoperative inflammatory and immune-nutritional markers and postoperative pulmonary complications after gastric and colorectal cancer surgery: a systematic review and narrative synthesis
Source: Front Surg. 2026 Jul 2;13:1850606. doi: 10.3389/fsurg.2026.1850606 (PMC13372747; doi:10.3389/fsurg.2026.1850606)
Supplement: Supplementary file 1 [file Supplementaryfile1.zip › Supplementary Table s1b.DOCX]

| Supplementary Table S1B. Detailed effect-extraction summary for the included studies | | | | | | |
| --- | --- | --- | --- | --- | --- | --- |
| Authors | Marker(s) with adjusted extractable data | Outcome selected for synthesis | Effect measure | Main adjusted association(s) extractable | Adjustment model / key covariates | Quantitative synthesis status |
| Chen et al. [10] | Albumin (also Hb and CRP screened) | PPCs within 30 days | Adjusted OR with 95% CI | Albumin (continuous): OR 1.15, 95% CI 1.06-1.28, p=0.011; AUC 0.728; cut-off 33.8 | Age, COPD, CHF, FEV1/FVC, operation duration, hemoglobin, albumin, CRP | Yes – directly poolable |
| Dai et al. [11] | RDW; SII (also NLR and PLR screened) | PPCs | Adjusted OR with 95% CI | RDW: OR 1.159 (1.025-1.309); SII: OR 1.001 (1.000-1.003) for PPCs | Age, RDW, SII in final model; NLR/PLR screened | Yes |
| Han et al. [12] | SIRI; AGR (also GNRI, PNI, NLR, PLR, SII, FPR, FAR screened) | Postoperative pulmonary infection (POI) during hospitalization | Adjusted OR with 95% CI | SIRI ≥0.773: OR 3.190 (1.401-7.261); AGR <1.58: OR 2.706 (1.186-6.174) | Age, respiratory disease, SIRI, AGR, operative method, operative duration; GNRI/PNI/NLR/SII/FAR/FPR screened | Yes – extractable from multivariable model |
| Inokuchi et al. [13] | Albumin (plus predicted VC as pulmonary variable) | PPCs and postoperative pneumonia | Univariate association only / no adjusted marker effect | Lower preoperative albumin significant in univariate only (p=0.023); not retained in final model | Sex, predicted VC, albumin, approach, extent of resection, operation time, bleeding, transfusion, pathologic stage | No – qualitative synthesis only |
| Kanno et al. [14] | Serum cholinesterase | Postoperative pneumonia (also infectious complications overall) | Adjusted OR with 95% CI | Serum cholinesterase independently associated with pneumonia (p=0.013); exact OR not visible in accessible text | Age, cerebrovascular comorbidities, cholinesterase, total gastrectomy, N stage | Yes |
| Kiuchi et al. [15] | Albumin / nutritional status | Postoperative pneumonia | Adjusted OR with 95% CI | Albumin <3.0 g/dL: OR 4.51; age ≥65: OR 3.59; stage ≥II: OR 2.35; hypertension: OR 2.21; total gastrectomy: OR 2.42 | Age, nutritional status, stage, hypertension, extent of gastrectomy | Yes |
| Li et al. [7] | CONUT score | PPCs and postoperative pneumonia | Adjusted OR with 95% CI | PPCs: mild vs normal OR 1.61 (1.18-2.20), moderate/severe vs normal OR 2.41 (1.51-3.84); pneumonia: 1.64 (1.07-2.52) and 2.51 (1.36-4.62) | Age, sex, heart disease, open surgery, blood transfusion, distant metastasis, colon vs rectum, CONUT category | Yes – directly poolable |
| Ma et al. [16] | PNI; PLR (also CONUT and NLR assessed) | Postoperative pulmonary infection (POI) within 30 days | Adjusted OR with 95% CI | PNI and PLR were independent risk variables for POI; exact ORs/95% CIs not visible in accessible text | Age, PNI, PLR, CA199, ASA score, postoperative ICU treatment; hemoglobin, albumin, NLR, CONUT screened | Yes |
| Mori et al. [5] | NLR (systemic inflammatory prognostic parameters screened) | Postoperative pneumonia | Adjusted OR with 95% CI | Preoperative NLR: OR 14.621 (1.160-184.348) for postoperative pneumonia | Clinical factors and systemic inflammatory-based prognostic parameters | Yes – directly poolable |
| Shoka et al. [6] | Systemic inflammation score (SIS) | Postoperative pneumonia | Adjusted OR with 95% CI | High SIS: unadjusted OR 3.10 (1.54-6.07); adjusted OR 2.31 (1.19-4.48), p=0.013; AUC 0.655 | 31 candidate variables in collaborative dataset | Yes – directly poolable |
| Sun et al. [8] | CONUT; GNRI; PNI | Postoperative pulmonary infection (PPI) | Adjusted OR with 95% CI | CONUT OR 2.23 (1.25-3.98); GNRI OR 0.94 (0.90-0.99); PNI OR 0.88 (0.81-0.97) | Age, mFI, CONUT, GNRI, PNI and clinical/surgical variables | Yes – directly poolable |
| Wu et al. [17] | Systemic immune-inflammation index (SII) | Postoperative pneumonia (POP) | Adjusted OR with 95% CI | Highest quartile preoperative SII: OR 6.017 (3.377-10.72) for POP | Demographics, labs including Hb/albumin/CRP, surgical and anesthesia duration, approach, pulmonary history | Yes |
| Xiang et al. [18] | Albumin | Postoperative pneumonia | Adjusted OR with 95% CI | Preoperative albumin was an independent predictor of pneumonia after CRC surgery (exact OR/95% CI not visible in accessible excerpt) | Age, sex, albumin, surgical method, surgical time | Yes |
| Zhang et al. [19] | Albumin (plus Hb and pulmonary function) | PPCs | Adjusted OR with 95% CI | Serum albumin <35 g/L was an independent risk factor for PPCs (p=0.004); exact OR/95% CI not visible in accessible text | Age, sex, FEV1/FVC, pulmonary disease history, hemoglobin, albumin, combined organ resection | Yes |
| Zhou et al. [20] | Total cholesterol (also albumin, CONUT, GNRI, PNI assessed) | PPCs | Adjusted OR with 95% CI | Total cholesterol: OR 0.75 (0.60-0.92) in training cohort; OR 0.62 (0.42-0.90) in validation cohort | Age >65, ASA, COPD, hemoglobin, albumin, total cholesterol, extent of resection, open surgery, duration, oxycodone dose | Yes |
| AGR, albumin-to-globulin ratio; ASA, American Society of Anesthesiologists; CA19-9, carbohydrate antigen 19-9; CONUT, controlling nutritional status; CRC, colorectal cancer; GC, gastric cancer; GNRI, geriatric nutritional risk index; NLR, neutrophil-to-lymphocyte ratio; OR, odds ratio; PNI, prognostic nutritional index; POP/POI/PPI, postoperative pneumonia/postoperative pulmonary infection; PPCs, postoperative pulmonary complications; PLR, platelet-to-lymphocyte ratio; RDW, red blood cell distribution width; SII, systemic immune-inflammation index; SIRI, systemic inflammation response index; SIS, systemic inflammation score. | | | | | | |
