# Supplementary material for: Preoperative inflammatory and immune-nutritional markers and postoperative pulmonary complications after gastric and colorectal cancer surgery: a systematic review and narrative synthesis
Source: Front Surg. 2026 Jul 2;13:1850606. doi: 10.3389/fsurg.2026.1850606 (PMC13372747; doi:10.3389/fsurg.2026.1850606)
Supplement: Supplementary file 1 [file Supplementaryfile1.zip › Supplementary Table s1a.DOCX]

| Supplementary Table S1A. Detailed study characteristics and pulmonary outcome definitions | | | | | | | | | | | | | | |
| --- | --- | --- | --- | --- | --- | --- | --- | --- | --- | --- | --- | --- | --- | --- |
| Authors | Year | Country | Study period | Setting / center | Study design | Cancer type / stage | Population / eligibility features | Total (n) | Pulmonary events, n (%) | Surgical procedure / approach | Preoperative marker(s) assessed | Timing of biomarker measurement | Comparator / coding strategy | Pulmonary outcome definition / time window |
| Chen et al. [10] | 2018 | China | NR | Ningbo No.2 Hospital | Single-center retrospective cohort | GC; stage NR | Elderly gastric cancer patients undergoing elective laparoscopic gastrectomy | 262 | 35 PPCs (13.4%) | Elective laparoscopic gastrectomy | Albumin (also Hb and CRP screened) | Preoperative laboratory variables | Continuous albumin; logistic regression; ROC analysis | PPCs within 30 postoperative days |
| Dai et al. [11] | 2022 | China | Jan 2019-Dec 2019 | Changhai Hospital, Naval Medical University | Single-center retrospective cohort | CRC; stage NR | Elderly patients receiving elective colorectal cancer surgery | 638 | 38 PPCs (5.96%) | Elective colorectal cancer surgery | RDW; SII (also NLR and PLR screened) | Preoperative inflammatory status assessment | Continuous markers with ROC-derived cut-offs | PPCs after elective colorectal surgery; overall incidence 5.96% |
| Han et al. [12] | 2025 | China | Jan 2017-Jun 2022 | First Hospital of Shanxi Medical University | Single-center retrospective study | CRC; stage NR | Adults undergoing radical colorectal cancer surgery | 866 | 72 POI (8.31%) | Radical CRC surgery | SIRI; AGR (also GNRI, PNI, NLR, PLR, SII, FPR, FAR screened) | Peripheral blood indexes within 1 week before surgery | Cut-offs from ROC/Youden; multivariable logistic regression and nomogram | CDC-based postoperative pulmonary infection during hospitalization |
| Inokuchi et al. [13] | 2014 | Japan | 1999-2011 | Single Japanese center | Single-center retrospective cohort | Gastric adenocarcinoma | Patients with gastric adenocarcinoma undergoing radical gastrectomy with lymphadenectomy | 1053 | 49 PPCs (4.7%) | Radical gastrectomy with lymphadenectomy | Albumin (plus predicted VC as pulmonary variable) | Preoperative factors | Univariate then multivariable logistic regression | PPCs within 30 days: pneumonia, macroscopic atelectasis, pneumothorax, ARDS |
| Kanno et al. [14] | 2024 | Japan | Jul 2014-May 2023 | Tokyo General Hospital | Single-center retrospective cohort | GC; stage/N stage reported | Patients undergoing gastrectomy for gastric cancer | 108 | 26 infectious complications; pneumonia count NR in accessible text | Gastrectomy for gastric cancer | Serum cholinesterase | Preoperative serum cholinesterase | Low vs high cholinesterase group; multivariable logistic regression | Postoperative pneumonia after gastrectomy; infectious complications also analyzed |
| Kiuchi et al. [15] | 2016 | Japan | 1997-2013 | Single Japanese center | Single-center retrospective cohort | GC; pStage analyzed | Consecutive patients undergoing curative gastrectomy for gastric cancer | 1415 | 31 postoperative pneumonia (2.2%) | Curative gastrectomy | Albumin / nutritional status | Preoperative clinical and laboratory assessment | Age ≥65; albumin <3.0; stage ≥II; hypertension; total gastrectomy | Postoperative pneumonia, Clavien-Dindo grade ≥II |
| Li et al. [7] | 2025 | China | NR | Sixth Affiliated Hospital of Sun Yat-sen University | Single-center retrospective cohort | CRC; distant metastasis included as covariate | Patients undergoing radical surgery for colorectal cancer | 2553 | 230 PPCs (9.0%) | Radical surgery for colorectal cancer | CONUT score | Preoperative CONUT score | Normal (≤1) vs mild malnutrition (2-4) vs moderate-to-severe malnutrition (≥5) | PPCs after radical CRC surgery; pneumonia reported separately |
| Ma et al. [16] | 2024 | China | Jan 2019-Dec 2021 | Lu'an Hospital, Anhui Medical University | Single-center retrospective cohort | GC; pTNM and pT/pN analyzed | Patients undergoing D2 radical gastrectomy for gastric cancer | 404 | NR in accessible text | D2 radical gastrectomy | PNI; PLR (also CONUT and NLR assessed) | Preoperative blood tests before treatment | ROC-derived cut-offs; multivariable logistic regression | POI within 30 days after surgery, diagnosed by CDC criteria |
| Mori et al. [5] | 2021 | Japan | NR | Single Japanese center | Single-center retrospective cohort | Stage I-III GC | Stage I-III gastric cancer patients after curative gastrectomy | 300 | Pneumonia count not isolated in accessible text; infectious complications 54/300 | Curative gastrectomy | NLR (systemic inflammatory prognostic parameters screened) | Preoperative systemic inflammatory parameters | Continuous/ROC-based inflammatory parameters; multivariable logistic regression | Postoperative complications classified as infectious/noninfectious; pneumonia most sensitive pulmonary outcome |
| Shoka et al. [6] | 2020 | Japan | 2010-2014 | Nine Japanese institutions | Multicenter retrospective cohort | GC; resected cohort | Patients who underwent gastrectomy for gastric cancer at 9 institutions | 1415 | 42 grade II or higher postoperative pneumonia (3.0%) | Gastrectomy with systematic lymph node dissection | Systemic inflammation score (SIS) | Preoperative SIS | High SIS vs lower SIS; optimal cut-off = 2 | Postgastrectomy pneumonia, grade II or higher |
| Sun et al. [8] | 2025 | China | Jan 2019-Jan 2025 | Affiliated Taizhou People's Hospital of Nanjing Medical University | Single-center retrospective cohort | CRC; TNM stage recorded | Elderly colorectal cancer patients undergoing radical resection | 339 | 40 PPI (11.8%) | Radical resection for colorectal cancer | CONUT; GNRI; PNI | Preoperative labs on the day before surgery | Continuous nutritional scores; multivariable logistic regression | PPI diagnosed by CDC/NHSN criteria |
| Wu et al. [17] | 2026 | China | Aug 2017-Aug 2023 | Two hospitals in Wuxi, China | Two-center retrospective cohort | CRC | Elderly patients undergoing colorectal cancer resection | 2500 | 171 POP (6.8%) | CRC resection | Systemic immune-inflammation index (SII) | Preoperative SII; postoperative SII change also analyzed | Highest quartile of preoperative SII (Q4) vs lower quartiles; PSM performed | Pneumonia within 30 days after surgery |
| Xiang et al. [18] | 2025 | China | Jan 2011-Dec 2021 | Single clinical center | Single-center retrospective cohort | CRC | Patients who underwent radical colorectal cancer resection | 7130 | NR in accessible text | Radical colorectal cancer surgery | Albumin | Preoperative albumin | Multivariable logistic regression; nomogram | Pneumonia after CRC surgery |
| Zhang et al. [19] | 2015 | China | Jan 2000-Dec 2009 | West China Hospital | Single-center retrospective cohort | GC | Gastric cancer patients with preoperative pulmonary function test undergoing gastrectomy | 685 (derived from 124 PPCs, 18.1%) | 124 PPCs (18.1%) | Gastrectomy | Albumin (plus Hb and pulmonary function) | Preoperative assessment | Albumin <35 g/L; multivariable analysis | PPCs after gastrectomy |
| Zhou et al. [20] | 2023 | China | Jan 2017-Dec 2021 | First Affiliated Hospital of Nanjing Medical University | Single-center retrospective cohort | GC | Gastric cancer patients undergoing elective gastrectomy | 2124 | 150 PPCs (7.1%) | Elective gastrectomy | Total cholesterol (also albumin, CONUT, GNRI, PNI assessed) | Preoperative laboratory parameters at admission | Continuous cholesterol; multivariable logistic regression in training and validation cohorts | In-hospital PPCs: pneumonia, pleural effusion, atelectasis, pneumothorax, respiratory failure |
| AGR, albumin-to-globulin ratio; ASA, American Society of Anesthesiologists; CA19-9, carbohydrate antigen 19-9; CONUT, controlling nutritional status; CRC, colorectal cancer; GC, gastric cancer; GNRI, geriatric nutritional risk index; NLR, neutrophil-to-lymphocyte ratio; OR, odds ratio; PNI, prognostic nutritional index; POP/POI/PPI, postoperative pneumonia/postoperative pulmonary infection; PPCs, postoperative pulmonary complications; PLR, platelet-to-lymphocyte ratio; RDW, red blood cell distribution width; SII, systemic immune-inflammation index; SIRI, systemic inflammation response index; SIS, systemic inflammation score. | | | | | | | | | | | | | | |
